# Rhythm production at school entry as a predictor of poor reading and spelling at the end of first grade

**DOI:** 10.1007/s11145-017-9782-9

**Published:** 2017-10-23

**Authors:** Kjersti Lundetræ, Jenny M. Thomson

**Affiliations:** 1 Norwegian Centre for Reading Education and Research, University of Stavanger, 4036 Stavanger, Norway; 2 University of Sheffield, Sheffield, UK

**Keywords:** Rhythm production, Early reading and spelling, Literacy difficulties

## Abstract

Rhythm plays an organisational role in the prosody and phonology of language, and children with literacy difficulties have been found to demonstrate poor rhythmic perception. This study explored whether students’ performance on a simple rhythm task at school entry could serve as a predictor of whether they would face difficulties in word reading and spelling at the end of grade 1. The participants were 479 Norwegian 6-year-old first graders randomized as controls in the longitudinal RCT on track (n = 1171). Rhythmic timing and pre-reading skills were tested individually at school entry on a digital tablet. On the rhythm task, the students were told to tap a drum appearing on the screen to two different rhythms (2 Hz paced and 1.5 Hz paced). Children’s responses were recorded as they tapped on the screen with their index finger. Significant group differences were found in rhythm tapping ability measured at school entry, when groups were defined upon whether children went on to score above or below the 20th percentile reading and spelling thresholds in national assessment tests at the end of grade one. Inclusion of the school-entry rhythmic tapping measure into a model of classification accuracy for above or below threshold reading and spelling improved accuracy of classification by 6.2 and 9.2% respectively.

**Electronic supplementary material:**

The online version of this article (doi:10.1007/s11145-017-9782-9) contains supplementary material, which is available to authorized users.

## Introduction

Children’s ability to read, especially in the primary years of education, remains a principle measure of an education system’s success. For individual children, their ability to read will help determine vocational options in the long term, while contributing to an individual’s sense of success at school in the immediate term. Yet learning to read is an incredibly demanding process for some children, leaving reading difficulties (RD) as the most frequent cause of special needs education in Norway, where this study is undertaken (Grøgaard, Markussen, & Hatlevik, 2004). More critically, children who display poor reading skills in their first year of formal reading instruction have been reported to have more than a 90% chance of continuing poor reading skills (Chard & Kameenui, 2000). Research and practice focused on trying to reduce this percentage converge on an understanding that early intervention is more effective at yielding gains in reading ability as compared to assistance offered in later school years (Torgesen, 2002; Vellutino et al., 1996). In turn, early intervention requires accurate screening and assessment tools that allow practitioners to effectively identify children at risk of reading difficulties, from the earliest stages of learning to read.

### Early identification of children at risk of reading difficulties

While a considerable amount of knowledge has amassed in understanding the relative predictive qualities of various literacy-related skills, we are still not at a point in any language where the identification of risk for reading difficulties in children starting their school careers can be carried out with certainty. For this reason, the current study sought to investigate additional measures that might have the potential to increase sensitivity of detection in a simple way. It also sought to use the strongest type of predictive design, a longitudinal study, which while not always possible, can offer more robust data than the more commonly used cross-sectional design. It is also important to note that children experiencing reading difficulties will also likely struggle in the parallel skill of spelling. While spelling has attracted less research attention, it requires more active, generative knowledge of written word forms and resultingly, is often a more persistent marker of literacy difficulties across alphabetic languages (Alegria & Mousty, 1994, 1996; Landerl, Wimmer, & Frith, 1997).

As soon as children have begun to acquire letter knowledge and are exposed to instruction in basic reading and spelling skills, these skills are themselves one of the most robust indicators of future reading potential (National Early Literacy Panel, 2008; Castles & Coltheart, 2004). However, before this stage, the picture is less clear. We know that children who have close family members with reading difficulties are at increased risk to develop reading difficulties themselves (Leavett, Nash, & Snowling, 2014; Pennington & Lefly, 2001). Also, across alphabetic languages, the ability to consciously reflect upon the composite sounds that combine to make whole words, a skill known as phonological awareness, has consistently been identified as an important early predictor of reading ability (de Jong & van der Leij, 2002; Storch & Whitehurst, 2002). Specifically, kindergarten and first grade children’s ability to identify and process the chunks of sounds—phonemes—that most commonly correspond to alphabet letters, has a well-documented relationship with early reading skills (see meta-analysis by Melby-Lervåg, Lyster, & Hulme, 2012). However, as noted by Scarborough (1998), when looking beyond general relationships and looking at phonological awareness’ (PA) ability to accurately *classify* children’s future reading status as either struggling, at-risk or superior, PA is a more successful predictor of future superior reading than of future reading problems. In other words, children who can demonstrate phonological sensitivity at or before formal reading instruction begins are unlikely to stumble later, whereas those identified as having weaker phonological sensitivity at school entry could very well go on to read satisfactorily.

Many of the early studies of reading predictors were carried out in English, an opaque orthography that takes significantly longer to master than more transparent alphabetic orthographies (Seymour, Aro, & Erksine, 2003). As knowledge about literacy acquisition in languages beyond English grows, evidence has emerged that the relative transparency of a language may have consequences for the types of pre-reading skill that best predict later reading success. One such contrast is the relative role of phonological awareness compared to rapid automatized naming, or RAN. For more transparent languages, given the faster pace of learning to read, the time window within which phonological awareness is a sensitive predictor of reading is potentially smaller than that for English (de Jong & van der Leij, 1999; Furnes & Samuelsson, 2010; Landerl & Wimmer, 2000; Leppänen, Niemi, Aunola, & Nurmi, 2006). In contrast, rapid naming appears to remain as a predictor of reading across grades in transparent orthographies (e.g. de Jong & van der Leij, 2003; van den Bos, Zijlstra, & Lutje Spelberg, 2002), while being more time-limited as a predictor in English (Parrila, Kirby, & McQuarrie, 2004; Roman, Kirby, Parrila, Wade-Woolley, & Deacon, 2009; Wagner et al., 1997). The current study is carried out in a Norwegian context, Norwegian being a relatively transparent language (Seymour, 2005). A recent longitudinal study by Furnes & Samuelsson (2011) that compared the relative power of RAN and phonological awareness in predicting reading and spelling in Norwegian/Swedish children compared to English-speaking children, across kindergarten and through Grades 1 and 2, found that RAN was related more to reading than spelling, while the opposite was true for phonological awareness, with similar patterns holding across languages. This study highlights the need to give separate consideration to predictors of reading versus spelling.

Oral language skills are another factor often considered in predicting early reading and spelling ability (e.g. Catts, Fey, Zhang, & Tomblin, 1999; Muter, Hulme, Snowling, & Stevenson, 2004). Oral language is an umbrella term that includes vocabulary knowledge, as well as the ability to understand and generate syntax and morphology. In studies of children acquiring literacy skills it is well established that oral language comprehension contributes significant and unique variance in predicting reading comprehension (Gough & Tunmer, 1986; Hoover & Gough, 1990). However, the role of oral language assessment in predicting early reading skills, when basic decoding skills are still being learned and consolidated, is less conclusive. For example, in an extensive meta-analysis of experimental or quasi-experimental studies looking at early prediction of reading and spelling ability, the National Early Reading Panel (Shanahan & Lonigan, 2010) concluded that simple measures of oral language, including measures of vocabulary, had a relatively weak relationship to both early decoding and spelling. More complex measures of oral language, for example grammar, definitional vocabulary and listening comprehension had stronger relationships, especially to early reading comprehension, though overall, the strength of these relations was not as consistently strong as more “code-based” predictors such as letter knowledge, rapid naming and phonological awareness.

### Music and language processing

Recently, increasing attention has been paid to the relationship between musical ability in children and their language and literacy skills (e.g. Cumming, Wilson, Leong, Colling, & Goswami, 2015; Gordon, Fehd, & McCandliss, 2015). Originating from isolated reports of musical training potentially conferring advantages in academic ability (Fisher, 2001; Hurwitz, Wolff, Bortnick, & Kokas, 1975), a field of study has now grown that is both exploring the wider impacts of music training in larger samples (Gordon, Fehd, & McCandliss, 2015), as well as trying to determine the causal mechanisms at play in links between music and oral/written language (Patel, 2011, 2014; Peretz, Vuvan, Lagrois, & Armony, 2015).

Regarding causal mechanisms, some of the most compelling research currently focuses upon neural overlap of music and language processing, and more specifically, the neural encoding of sound (Goswami, Power, Lallier, & Facoetti, 2014; Kovelman, Mascho, Millott, Mastic, Moiseff, & Shalinsky, 2012; Kraus & Slater, 2015). Sound processing is an integral aspect of music perception (Angulo-Perkins & Concha, 2014), while highly developed sound processing is also needed in order to learn the sound-letter correspondences that are the foundation of literacy across alphabetic languages (Melby-Lervåg, Lyster, & Hulme, 2012). Converging findings from different research groups (e.g. Goswami, 2011; Tierney & Kraus, 2014) highlight the potential importance of rhythm perception in music and speech relations. Developmentally, speech rhythm is one of the first cues used by infants to segment the speech stream into words and word parts (Ramus, Hauser, Miller, Morris, & Mehler, 2000; Eimas, Siqueland, Jusczyk, & Vigorito, 1971) and caregivers naturally exaggerate the rhythm of their speech when interacting with their infants (Fernald et al., 1989). Spoken syllables provide language with a regular beat—within words syllables carry core information regarding a word’s sound, or phonological structure, as well as its morphology (Hayes, 1995), whilst the combination of syllables and words into another aspect of speech rhythm—intonation—contributes to meaning. It has been found that children with specific language impairments have difficulties in speech (Richards & Goswami, 2015) and non-speech rhythm perception (Corriveau, Pasquini, & Goswami, 2007) as well as difficulties in tapping in time to (non-speech) beat (Corriveau & Goswami, 2009). It has also been demonstrated that children and adults who struggle to learn to read have difficulty in beat perception (Hämälainen, Salminen, & Leppänen, 2013; Leong & Goswami, 2014) and production (Thomson & Goswami, 2008) across both speech (Goswami, Gerson, & Astruc, 2010; Wood & Terrell, 1998) and non-speech (Overy et al., 2004; Wolff, 2002) domains. Difficulties in beat perception and production are likely to be particularly important for learning to decode, a reading subskill reliant on intact phonological abilities. Emerging evidence also points to links between basic rhythm skills and more advanced reading skills, including reading comprehension (Goswami, Huss, Mead, Fosker, & Verney, 2013), however, our understanding of these relationships remains more limited.

Interest in the potential importance of beat perception/production at the earliest stages of learning to read has precipitated an emerging body of empirical evidence that examines beat perception or production in preschool children or children at the age of school-entry and its relationship to reading-related skills, largely in the domain of non-speech beat perception. Woodruff Carr, White-Schwoch, Tierney, Strait & Kraus (2014) studied a sample of thirty-five English speaking children between the ages of 3 and 4, using a drumming paradigm in which children were encouraged to tap on a drum in synchrony with an experimenter, whose drum rate approximated to a syllabic rate of presentation (1.67 and 2.5 Hz). Woodruff Carr et al. found that the children who were better able to synchronize or entrain to the external beat had significantly superior phonological processing (a task including compound word and syllable blending, sentence and syllable segmentation, rhyme awareness and production), auditory short-term memory (recalling sentences), and rapid naming ability (colours and objects) compared to their peers who were less able to synchronize to the beat. The authors also found significant neurophysiological differences between the two groups, as measured by auditory brainstem responses (ABRs), proposing a causal relationship between neural temporal sensitivity and reading readiness.

With children at the very start of their school careers, in Kindergarten, relationships between non-linguistic beat sensitivity and reading-related skills has also been reported. Corriveau, Goswami, & Thomson (2010), for example, explored relationships between auditory rise-time perception and reading related skills, including letter-sound knowledge, syllable, rhyme and phonemic awareness, in a group of 88 3- to 6-year old English-speaking children. Rise time is a dynamic measure of the time taken for a sound to reach its maximum amplitude and correlates to the perception of a beat in non-linguistic sounds, while correlating to the point of peak amplitude or loudness in a speech syllable (Scott, 1998). In this study, sensitivity to rise time, in contrast to other auditory variables (including frequency and intensity) was found to be a significant predictor of early reading-related skills in both a longitudinal and cross-sectional analysis, particularly for the development of rhyme awareness skills.

In addition, a study by Moritz, Yampolsky, Papadelis, Thomson & Wolf (2013), looked at both cross-sectional and longitudinal predictors of reading and related skills in a group of 30 US kindergartners (5 year-olds), twelve of whom were followed up at the end of second grade (mean age: 8 years, 1 month). The study included three measures of rhythm ability. One measure tested rhythm discrimination using a same-different judgment paradigm while there were two measures of rhythm production: tempo copying, which involved copying four drum beats presented at an even tempo, with different trials varying in inter-onset interval, and rhythm copying, which involved copying short rhythmic sequences of 3–7 patterned beats. In both the cross-sectional and longitudinal analyses the productive measures demonstrated significant relationships with phonological awareness and reading itself (the latter only possible to measure in the longitudinal analysis). A final study of note is of Dellatolas, Watier, Le Normand, Lubart, and Chevrie-Muller (2009). 1028 French kindergartners aged 5–6 years carried out a 21-item rhythm copying task and when 695 of the group were followed up in second grade the rhythm copying task administered in kindergarten had a strong, linear relationship to reading performance in second grade.

A developing literature thus supports the hypothesis of a link between non-linguistic rhythm sensitivity and emerging literacy skills in pre-school children or those just beginning to be exposed to formal literacy instruction. However, significant questions remain. Firstly, all of the studies reported above have been carried out in English language contexts. Languages differ in both their rhythmic structure (Peppé et al., 2009) as well as the transparency of the relationship between speech sounds and written symbols (Ziegler & Goswami, 2005), which may have implications for the strength of relationships between children’s rhythm skills and their literacy development across languages. Studies by Goswami and colleagues suggest a degree of language-universality in the relationship (see Goswami & Leong, 2013 for an overview), however these findings are not always replicated (e.g. Georgiou, Protopapas, Papadopoulos, Skaloumbakas, & Parrila, 2010). Secondly, it remains to be seen which specific measures of beat perception or production are the optimal predictors of reading-related skill.

### Research aims

In this study, our aim was to study whether measurement of children’s production of rhythm has the potential to increase the accuracy of prediction of reading and spelling difficulties in Norwegian 6-year-old first graders, and if so, to what degree. Specifically, we were interested in whether students’ performance on a simple rhythm task at school entry could serve as a unique predictor of difficulties in word reading and spelling at the end of grade 1. In Norway, literacy instruction starts at the beginning of the first grade and national tests administered at the end of grade one are used to identify risk for reading and writing difficulties, based on a performance in the bottom 20th percentile of a national norm sample. However, given the value of early intervention discussed at the beginning of this section, having a range of pre-literacy measures that could accurately assess risk at the point of school entry would allow for intensified support as soon as children start learning to read, thus reducing experiences of struggle or failure, and the size of the ability gap. A further advantage of measuring rhythm ability at school entry is the opportunity this provides to measure the skill before children have been exposed to formal music training. Tsang and Conrad (2011) found that rhythm discrimination was significantly enhanced in young children (aged between 5 and 9 years old) who had received approximately 2 years of music training, in comparison to a group with no formal instruction. Further, the authors found that relationships between rhythm discrimination and reading-related skills, including phonological awareness and single word reading, were significantly different in the groups, with stronger correlations present in the non-trained group. In Norway, formal music instruction before school entry is very rare, which allowed us to examine untrained rhythm aptitude in the first instance.

It was decided to use a measure of beat production, modelled on the task used by Thomson & Goswami (2008) and similar to that used by Woodruff Carr et al. (2014) and Moritz et al. (2013). Obtaining reliable results from auditory *perceptual* tasks in young children can be a challenge (e.g. Abeele, Wouters, Ghesquière, Goeleven, & Geurts, 2015) and a pragmatic aim of this study was that the measures used would be feasible for large-scale usage within a test battery that was as time-efficient as possible. We thus decided to utilize the precedent of a beat *production* task, as reported in the studies above. This was administered alongside a broader battery of pre-reading measures including letter knowledge, phonemic awareness, short-term memory, RAN and vocabulary; the inclusion of these measures would allow the predictive contribution of rhythm ability to be compared to skills previously investigated as predictive variables.

## Methods

### Participants

The participants were 479 Norwegian 6-year-old first graders randomized as controls in the longitudinal RCT On Track (n = 1171) (Lundetræ, Solheim, Schwippert, & Uppstad 2017). The On Track sample is a convenience sample of 19 primary schools within close traveling distance of the University of Stavanger, and was recruited during the spring of 2014. The schools met the following two conditions: (1) more than 40 children were expected to be enrolled in grade 1 in 2014; and (2) the school’s score on the national reading tests had been close to the national mean (2.0 ± 0.1 on a scale from 1 to 3) in two of the three previous years. 97.7% of the children got their parents’ consent for participation. Children with reported hearing difficulties, as identified by parent report, were excluded from the sample (n = 12). Gender was almost equally distributed across the sample, with 53.7% girls and 46.3% boys. 13.2% of the children had no parents speaking a Scandinavian language at home, and 18.4% had a mother and/or father who self-reported reading and writing difficulties (RWD). 61.6% of parents in the sample held a higher degree. In Norway as a whole 47.2% of 30–39-year olds hold a higher degree, however in larger cities and municipalities, such as the location of this study, the rate is typically higher (for example, in the capital, Oslo, the same percentage is 62.3%) (Statistics Norway, 2017).

### Procedure

All children were assessed individually during the 4 weeks just after the school entry of 2014. The testing was administered in 20–30-min sessions during school hours in a quiet room at the child’s school. The testers were all experts in the field of reading education and individual testing, and had received 6 h of training in the administration of the specific test battery. The test battery was administered on a Lenovo Yoga Tablet 10, Android 4.2, and for all tests, student responses were scored and recorded on the tablet. The test results from the tablets were generated and stored as Microsoft Excel files. All students completed all tests, with no missing data. In addition, parents answered a questionnaire relating to demographics, home literacy environment, familial risk of RWD, the student’s language background, and his or her health. The response rate for the parental questionnaire was 97.5%.

In the end of grade 1, literacy tests were pen- and paper based and administered in small groups in two 45–60-min sessions. The children were seated at separate tables, with a distance not allowing for copying the answers from one another. Trained testers administered the assessments in a fixed order to all children.

### Measures

#### At school entry

##### Rhythmic tapping ability

Rhythmic tapping ability was measured by means of the On Track Rhythm Test (Lundetræ, 2015) in a manner based on work described by Thomson and Goswami (2008). A drum appeared on the screen, and the students were told that they would hear a rhythm and that they should tap the drum to the rhythm. When they started hearing a new rhythm, they should tap to that new rhythm. Each metronome speed was presented for 30 s (paced), using an 800 Hz pure tone of 10 ms’ duration. The task lasted for a total of 1 min, and the blocks of sounds were presented in the following order: 2 Hz paced, 1.5 Hz paced. The students’ responses in the form of tapping on the screen with their preferred index finger were recorded. Prior to the actual test, all students completed a practice block lasting for 20 s, with 10-second blocks of 2 and 1.5 Hz, respectively. The time between taps—the inter-tap interval (ITI)—was calculated as the difference between two subsequent responses (response 2 minus response 1 (ITI 1), response 3 minus response 2 (ITI 2), etc.). An ITI of 500 ms (2 Hz) or 666.7 ms (1.5 Hz) indicates tapping at exactly the same pace as the metronome’s beeping. ITI was calculated for both rhythm speeds (1.5 and 2 Hz), and outliers (e.g. where children skipped to tap to a beep) were removed if the ITI fell outside 3.27 standard deviations of the group mean (90% confidence interval). In line with Corriveau & Goswami (2009), we created a measure of the extent to which each child produced appropriate ITIs by calculating the absolute value ITI difference scores. For the 2 Hz, the target ITI was e.g. 500 ms, and a mean ITI of 480 would correspond to an ITI difference score of (480–500 ms) = 20 ms. Reliability (Cronbach’s alpha) was .95 for the 2 Hz rate and .96 for the 1.5 Hz rate, and children’s performance on the two measures correlated *r* = 0.55 (*p* < 0.01).

##### Letter-sound knowledge

Letter-sound knowledge was measured using a 15-item multiple-choice test. The stimulus was a pre-recorded letter sound, and the student’s task was to identify the corresponding upper-case letter. The student responded by pressing one of four letters appearing on the screen. Reliability (Cronbach’s alpha) was .84.

##### Phonemic awareness

Phonemic awareness was measured by means of eight first-phoneme-isolation and eight phoneme-blending items. Both subtests included two practice items. The phoneme-isolation items consisted of a picture. The tester pointed to the picture, named the object, and asked the student to give the first sound, for instance by saying, “This is an/eple/[English: ‘apple’]. What is the first sound in/eple/?” The student responded orally. The test was automatically discontinued if a student made two subsequent errors. Reliability (Cronbach’s alpha) was .93.

The phoneme-blending items consisted of four pictures. The target stimuli was prerecorded to ensure that the phonemes would be presented in an identical manner to each student with regard to pronunciation and time interval: “Here you see pictures of/ri/,/rips/,/ris/, and/ring/[English: ‘ride’, ‘redcurrant’, ‘rice’, ‘ring’]. Listen carefully and press the picture that goes with:/r//i//s/[presented phoneme-by-phoneme, one phoneme per second].” The eight test items were ordered by difficulty (easiest first), based on the results of a pilot test, and was automatically discontinued if a student made two subsequent errors. Reliability (Cronbach’s alpha) was .87.

##### RAN (rapid automatized naming)

RAN was measured through timed naming of familiar objects presented simultaneously in random order in a left-to-right serial. The objects were ‘sun’, ‘car’, ‘plane’, ‘house’, ‘fish’, and ‘ball’, all of which are monosyllabic words in Norwegian. There were four rows of five objects in each matrix, and a different matrix was used for each of two trials. The student was asked to name each object as quickly and accurately as possible, working from left to right and from top to bottom. During a practice session, the tester made sure that the student could name all the objects in the matrix and understood the task. For each trial, the time required to complete the task (in 1/100ths of a second) and any naming errors were recorded.

##### Short-term memory (STM)

STM was measured using the Digit Span Forward test from the third edition of the Wechsler Intelligence Scales for Children (Wechsler, 1991). The stimuli were read aloud by the tester at a rate of one digit per second, and the student’s responses were scored on the tablet. The test was automatically discontinued after two subsequent errors.

##### Vocabulary

Vocabulary was measured using an abridged version (20 of the original 40 items) of the Norwegian Vocabulary Test (NVT), which is designed for children aged 5–6 (Størksen, Ellingsen, Tvedt, & Idsøe, 2013). For each item, a picture appeared on the screen and the students were asked to name it. Reliability (Cronbach’s alpha) for the 20 items was .83, which is consistent with the Cronbach’s alpha value for the full 40-item test in the original standardization sample (.84).

##### RWD in family

Information about reading and writing difficulties in the family was derived from the parents’ questionnaire. The item “Has anyone in the child’s biological family experienced reading and writing difficulties?”, had the following separate response alternatives for mother and father: ‘yes’, ‘no’, ‘don’t know’. If it was checked ‘yes’ for mother and/or father, the variable *RWD in family* was scored 1. If it was checked ‘no’ or ‘don’t know’ for both, the variable was scored 0.

#### End of first grade

To identify struggling readers, we used national cut-offs for word reading and spelling as reported in the test manual for the Norwegian National assessment test for end of grade 1 (The Norwegian Directorate for Education and Training, 2015). The National assessment tests are devised to identify children at risk for reading and writing difficulties. If children perform below the 20th percentile on one of the subtests (the national threshold), steps should be taken by a school in order to enhance the student’s literacy skills. Hence, the tests are not normally distributed, but provide the teachers with detailed information on the 40% lowest performing students.

##### Word reading

The word-reading subtest from the Norwegian National assessment test for grade 1 consisted of 14 items, and the time limit was 5 min. Each item consisted of a picture followed by four visually similar words, whereof one corresponded to the picture. Following a practice item, the child was asked to read the words as fast as possible and to check the word that matched the picture. E.g. a picture of a fish (‘fisk’ in Norwegian) followed by ‘fiske’, ‘fikse’, ‘fiks’ and ‘fisk’. The correct stimuli was presented in a random order. Number of correct words was measured (maximum = 14). Struggling readers were defined as those who fell below the national threshold of reading 9 words or less correct (13.4% of the sample).

##### Spelling

The spelling subtest from the National assessment test for grade 1, consisted of 14 items. For all items, the tester first read a short sentence containing the target word, for instance, “It was a difficult test. Write ‘test’”. Number of orthographic correct spelled words was measured (maximum = 14). Struggling spellers were defined as those who fell below the national threshold of spelling 8 words or less correct (13.6% of the sample).

## Results

Table 1 shows descriptive statistics for predictor measures at start of grade 1. It is worth noting that most children knew many letter-sounds before the onset of reading instruction, and that identification of first phoneme had a ceiling effect. A table of correlations between predictors at the start of grade 1 is also given in the Supplementary material, Appendix A.

**Table 1:** Descriptive statistics for predictor measures at start of grade 1

	Mean	SD	Min.–max.
Letter knowledge	12.03	3.28	0–15
First phoneme isolation	5.30	2.98	0–8
Phoneme blending	3.49	2.65	0–8
Vocabulary	12.81	4.01	1–20
RAN	61.58	15.39	29.71–142.72
STM	5.41	1.48	0–11
ITI diff. 2 Hz	48.32	73.60	0.00–301.86
ITI diff. 1.5 Hz	102.56	130.05	0.00–451.07

*STM* short-term memory,* ITI* inter-tap interval

A series of t-tests were conducted to investigate group differences in emergent literacy skills and rhythmic tapping ability at school entry between children with and without difficulties in word reading and spelling at the end of grade 1. Significant group differences in emergent literacy skills and rhythmic tapping were found between children below and above the national threshold in word reading (Table 2) and spelling (Table 3). Large effect sizes were found for Letter Knowledge and First Phoneme Isolation at school entry, regarding the discrepancy in performance between good and poor readers as identified at the end of grade 1. Medium effect sizes were found for Phoneme Blending, RAN, and rhythmic tapping ability at the 2.0 and 1.5 Hz rate (see Table 2) for the same comparison.

**Table 2:** Mean scores at school entry for students with word reading scores below (n = 64) and above (n = 415) the national threshold at the end of grade 1

	Reading below national threshold	(SD)	Reading above national threshold	(SD)	t(477)	*p*	Cohen’s d
Letter Kn.	9.14	(3.65)	12.48	(2.98)	6.96	<.001	1.00
First Phon.	2.28	(2.75)	5.76	(2.73)	9.48	<.001	1.27
Blending	1.86	(2.03)	3.74	(2.64)	6.59	<.001	0.80
Vocabulary	10.56	(4.32)	13.16	(3.85)	4.94	<.001	0.64
RAN	72.99	(19.02)	59.82	(13.97)	− 5.32	<.001	− 0.79
STM	4.53	(1.30)	5.55	(1.46)	5.26	<.001	0.74
ITI dif. 2 Hz	86.44	(94.06)	41.95	(67.83)	− 3.64	<.001	− 0.54
ITI dif. 1.5 Hz	174.37	(158.96)	91.49	(121.50)	− 4.00	<.001	− 0.59

*SD* standard deviation,* STM* short-term memory,* ITI* inter-tap interval

**Table 3:** Mean scores at school entry for students with spelling scores below (n = 65) and above (n = 414) the national threshold at the end of grade 1

	Spelling below national threshold	(SD)	Spelling above national threshold	(SD)	t(477)	*p*	Cohen’s d
Letter Kn.	9.02	(3.56)	12.50	(2.97)	7.51	<.001	1.06
First Phon.	1.75	(2.29)	5.86	(2.68)	13.09	<.001	1.65
Blending	1.45	(1.89)	3.81	(2.61)	8.85	<.001	1.04
Vocabulary	9.92	(4.16)	13.27	(3.80)	6.52	<.001	0.84
RAN	70.70	(19.37)	60.15	(14.18)	− 4.22	<.001	− 0.62
STM	4.35	(1.18)	5.58	(1.45)	7.52	<.001	0.93
ITI dif. 2 Hz	88.62	(92.67)	41.50	(67.78)	− 3.94	<.001	− 0.58
ITI dif. 1.5 Hz	185.41	(155.23)	89.71	(120.91)	− 4.70	<.001	− 0.69

*SD* standard deviation,* STM* short-term memory,* ITI* inter-tap interval

Carrying out the same analysis for children identified as good or poor spellers at the end of grade 1, large effect sizes were found between these groups regarding First Phoneme Isolation, Letter Knowledge, Phoneme Blending, Short Term Memory, and Vocabulary performance, while medium effect sizes were found for RAN, and rhythmic tapping ability at the 2.0 and 1.5 Hz rate (see Table 3).

For both word reading and spelling, larger effect sizes were found for rhythmic tapping ability at the 1.5 Hz (667 ms) rate than for the 2 Hz (500 ms) rate. For this reason, the rhythmic tapping ability at the 1.5 Hz rate was used as a predictor in the multivariate logistic regression analyses for prediction of difficulties with word reading or spelling at the end of grade one (i.e. performing below the national thresholds). In addition, gender, family risk for reading and writing difficulties, short term memory, vocabulary, letter knowledge, first phoneme identification, phoneme blending, and RAN at school entry served as predictors in the analyses displayed in Tables 4 and 5. In these analyses, all predictor variables were entered simultaneously, but unique variance was calculated for each predictor. The logistic regression analyses provided models that fitted the data well, *χ* ^2^ (9, *N* = 479) = 114.73, *p* < .001, for Word Reading, and *χ* ^2^ (9, *N* = 479) = 156.11, *p* < .001, for Spelling, and explained 39.1 and 50.7% of the variance (Nagelkerke R^2^) in difficulties in Word Reading and Spelling, respectively.

**Table 4:** Logistic regressions of word reading below the national threshold at the end of grade 1

	B	s.e. B	OR	95% CI	R^2^
Girl	− 0.504	0.329	0.604	(0.317–1.151)	0.7
RWD in fam.	0.710	0.358	2.035*	(1.009–4.105)	1.1
STM	− 0.272	0.138	0.762*	(0.581–0.998)	1.2
Vocabulary	0.012	0.045	1.012	(0.926–1.105)	0.0
Letter Kn.	− 0.148	0.050	0.863**	(0.783–0.951)	2.6
First sound	− 0.239	0.068	0.787***	(0.689–0.899)	4.1
Blending	− 0.015	0.085	0.985	(0.834–1.163)	0.0
RAN	0.032	0.010	1.032**	(1.012–1.053)	3.0
ITI dif. 1.5 Hz	0.002	0.001	1.002^a^	(1.000–1.004)	1.0

*RWD* reading and writing difficulties,* STM* short-term memory,* ITI* inter-tap interval

* *p* < .05; ** *p* < .01; *** *p* < .001; ^a^ *p* = 0.067

**Table 5:** Logistic regressions of Spelling below the national threshold at the end of grade 1

	B	s.e. B	OR	95% CI	R^2^
Girl	− 0.469	0.352	0.626	(0.314–1.246)	0.4
RWD in fam.	1.357	0.381	3.886***	(1.840–8.207)	3.5
STM	− 0.456	0.155	0.634**	(0.468–0.860)	2.6
Vocabulary	− 0.039	0.048	0.962	(0.876–1.057)	0.1
Letter Kn.	− 0.125	0.056	0.882*	(0.791–0.984)	1.3
First sound	− 0.323	0.077	0.724***	(0.623–0.842)	5.8
Blending	− 0.174	0.101	0.840	(0.689–1.025)	0.8
RAN	0.011	0.011	1.011	(0.989–1.034)	0.2
ITI dif. 1.5 Hz	0.002	0.001	1.002*	(1.000–1.005)	1.1

*RWD* reading and writing difficulties,* STM* short-term memory,* ITI* inter-tap interval

* *p* < .05; ** *p* < .01; *** *p* < .001

For reading below the national thresholds, the following predictors were significant: familial risk for RWD, short term memory, letter knowledge, first phoneme identification, and RAN. For rhythmic tapping ability there was a statistical trend toward significance (*p* = 0.067). For spelling below the national thresholds, the following predictors were significant: familial risk for RWD, short term memory, letter knowledge, first phoneme identification, and rhythmic tapping ability.

Short-term memory, letter knowledge, and first phoneme identification yielded negative b-values and the odds ratio for group differences in word reading and spelling was below 1. That is, the higher the scores on these measures at school entry, the less likely it is that a child would struggle with reading or spelling at the end of grade 1. Home language was not included in the logistic regressions as it did not add anything beyond vocabulary.

We show in Table 6 that a model including gender, family risk for RWD, STM, vocabulary, letter knowledge, phonemic awareness, and RAN identified 29.7% of prospective poor readers and correctly classified 89.1% of students overall. When including rhythmic tapping ability in the next block, accuracy of identification of poor readers improved by 6.2 percentage points, while overall classification accuracy was improved by 0.5 percentage points. The group of correctly identified poor readers therefore increased from 19 to 23 based on the inclusion of rhythmic tapping ability. 71.9% of those who were predicted to be poor readers were ultimately categorized as poor readers at the end of grade 1, while 90.1% of those who were predicted not to have difficulties in reading were correctly classified.

**Table 6:** Prospective classification of children into below or above national thresholds in word reading at the end of grade 1 based on models without and with a measure of rhythmic tapping ability (1.5 Hz)

	Observed (end of grade 1)	Predicted reading		Correct identification rate	Overall correct identification rate
		Below national threshold	Above national threshold		
Gender, family risk for RWD, STM, Voc., LK, first phoneme identification, blending, RAN	Above threshold	7	408	98.3	
	Below threshold	19	45	29.7	89.1
Gender, family risk for RWD, STM, Voc., LK, first phoneme identification, blending, RAN, rhythm	Above threshold	9	406	97.8	
	Below threshold	23	41	35.9	89.6

*Voc.* vocabulary, *STM* short-term memory, *LK* letter knowledge

Table 7 shows that a model including gender, family risk for RWD, STM, vocabulary, letter knowledge, phonemic awareness and RAN identified 40% of prospective poor spellers and correctly classified 89.1% of students overall. When including rhythmic tapping ability, classification accuracy of poor spellers improved by 9.2% points for sensitivity, and by 1.1% points across all students. Inclusion of rhythmic tapping in the model therefore increased the group of correctly identified poor spellers from 26 to 32 students. 69.6% of those who were predicted to be poor spellers were categorized as poor spellers at the end of grade 1, while 92.4% of those who were predicted not to have difficulties in spelling were correctly classified.

**Table 7:** Prospective classification of children into below or above national thresholds in spelling at the end of grade 1 based on models without and with a measure of rhythmic tapping ability (1.5 Hz)

	Observed (beginning grade 1)	Predicted spelling		Correct identification rate	Overall correct identification rate
		Below national threshold	Above national threshold		
Gender, family risk for RWD, STM, Voc., LK, first phoneme identification, blending, RAN	Above threshold	13	401	96.9	
	Below threshold	26	39	40.0	89.1
Gender, family risk for RWD, STM, Voc., LK, first phoneme identification, blending, RAN, rhythm	Above threshold	14	400	96.6	
	Below threshold	32	33	49.2	90.2

*Voc.* vocabulary, *STM* short-term memory, *LK* letter knowledge

## Discussion

In this study we sought to investigate whether measurement of children’s rhythm skills had the potential to increase the accuracy of detection of reading and spelling difficulties in Norwegian 6-year-old first graders. Specifically, we were interested in whether students’ performance on a simple rhythm task at school entry could serve as a predictor of poor abilities in word reading and spelling at the end of grade 1. We found significant group differences in children’s ability to tap in time to an externally-delivered beat, measured at school entry, when groups were defined upon whether children went on to score above or below the 20th percentile threshold in national assessment tests at the end of grade one.

Regarding statistical prediction of reading and spelling at the end of grade one, using logistic regressions we found that children’s rhythmic tapping ability at the 1.5 Hz rate was a significant predictor of group membership to the below-threshold spelling group, alongside short-term memory, letter knowledge, first phoneme identification and familial risk for RWD. For spelling below the national threshold, the correct identification rate increased from 40.0 to 49.2. Spelling difficulties are found to be more persistent than difficulties with reading accuracy in dyslexia (Hulme & Snowling, 2009). Hence, an increase in the correct identification rate of spelling below the national threshold of 9.2% points based on a simple measure of rhythm constitutes a significant contribution to early identification of children at risk for RWD.

Children’s rhythmic tapping ability at the 1.5 Hz rate fell short of significance (0.07) as a predictor of group membership to the below-threshold reading group, while letter knowledge, first phoneme identification, RAN, and familial risk for RWD were significant predictors. However, when looking at classification accuracy, the correct identification rate of students reading below the threshold at the end of grade 1 increased from 29.7 to 35.9% when rhythmic tapping was included in the model.

The findings of Moritz et al. (2013) share a longitudinal design with the current study, exploring relationships between receptive and expressive rhythm measures in Kindergarten (when the children were starting to be exposed to formal literacy instruction, similar to the children at school entry in this study), with reading at the end of second grade. In a group of just twelve children, significant relationships were found between rhythm pattern copying in kindergarten and reading in second grade (spelling was not measured in this study). On the surface, the tempo copying task used by Moritz et al. appears more similar to the measure used in this study, in that children were asked to copy a regular string of beats in the tempo copying task, as in the drum beat task here. The rhythm copying task, in contrast, required copying of rhythm patterns where sounds were presented in a mixed-interval sequence. However, the tempo copying of Moritz involved the presentation of just four sounds, at an even interval, to be copied subsequently by the child. Here, children were trying to tap in synchrony with an ongoing beat, for 30 s per speed. The Moritz et al. tasks therefore involve memory and motor repetition, while the task in the current study requires synchronous beat entrainment. In this regard, the task is closer to that used cross-sectionally by Woodruff Carr et al. (2014) with 3–4 year olds. In the Woodruff Carr et al. study, while it was too early to assess reading per se, the findings again complement those reported here, in that children’s accuracy in synchronizing to a beat associated significantly with pre-reading skills, as measured through phonological awareness, auditory short-term memory and rapid naming.

Relationships between rhythm processing and spelling have been less consistently studied in comparison to the relationship between rhythm processing and reading. In a now-seminal study by Katie Overy that looked at the effects of a 15 week rhythm-based intervention for children with dyslexia (mean age 8.8 years), the intervention resulted in significant gains in spelling, but not reading ability (Overy, 2000). The children also made significant gains in rhythm copying and phonological ability. Overy was cautious in interpreting the relatively greater gains in spelling versus reading, noting that progress in reading may have followed the initial spelling and phonological increases (the study was not able to follow-up the children’s progress longer term). However, Overy also noted Frith’s hypothesis (Frith, 1985) that phonological awareness and spelling are particularly tightly coupled in early literacy learning. Indeed, Frith argues that the alphabetic principle—understanding that there are systematic and predictable relationships between letters and sounds—typically emerges first in children’s writing, as opposed to their reading. Thus, if we posit that rhythm processing will most likely influence literacy development through its contribution to phonological development, then it may be that the relative strength of relationships between rhythm and spelling/reading will change during the course of a child’s literacy acquisition, dependent upon the particular phonological demands present. This proposition might in turn partly explain the mixed results of cross-sectional studies that have looked at predictive relationships between rhythm processing and spelling. The predictive strength has sometimes been stronger than that of reading (Thomson & Goswami, 2008), sometimes approximately equal (e.g. Goswami, Gerson, & Astruc, 2010), and sometimes less (e.g. Holliman et al., 2017; Huss, Verney, Fosker, Mead & Goswami, 2011). It is likely, however, that differences in assessment measures (e.g., as well as the presence/absence of literacy difficulties or risk thereof) will also contribute to this mixed picture of findings.

To our knowledge this is the first reported use of a non-linguistic beat-based test used in the prediction of early literacy skills in a language other than English. Norwegian and English are both Germanic languages and so share in common many phonological, morphological and prosodic features. Considering rhythm and literacy specifically, rhythmically, a significant shared prosodic characteristic between Norwegian and English are their predominantly trochaic syllable rhythms of strong stress on the first syllable of the word (Jusczyk, Friederici, Wessels, Svenkerud, & Jusczyk, 1993). Orthographically, while both language are alphabetic, Norwegian contrasts with English in being more transparent, resulting a slightly different pathway to literacy competence for Norwegian and English children—English children can rely slightly less on the constancy of letter-sound correspondences and have to learn to recognize larger chunks of words in order to find consistency (Ziegler & Goswami, 2005). The results here suggest that for two languages with similar rhythms but different orthographic characteristics, the predictive significance of rhythm performance remains common to both. It is also important to note that the overall variance in reading and spelling explained by the regression models in this study (39.1 and 50.7% respectively) is very consistent with existing studies in both English and Scandinavian languages. Heath & Hogben (2004, p. 752) note that “the amounts of variance in reading achievement accounted for so far have typically varied between 40 and 60%”, while logistic regression models in Furnes and Samuelsson’s study (Furnes & Samuelsson, 2010) with a Scandinavian sample accounted for 36 and 48% of variance in grade 1 reading and spelling respectively, strikingly similar results, also using dichotomous variables. Additional variance in this study as well as its predecessors could be accounted for e.g. the quality of literacy instruction provided during first grade, genetics as well as the home literacy environment.

In situating the current findings in relation to existing research, another question pertains to the profile of children identified as “poor readers” and whether these individuals have similar characteristics across studies. In the first year of schooling, the main task of learning to read is developing an ability to decode and identify words and it is only in the later grades that the demands of reading comprehension become more salient (Adams, 1994). In turn, across studies that identify struggling readers in the earliest stages of learning to read, difficulties in single word reading and spelling are a common trait (e.g. Boscardin, Muthén, Francis, & Baker, 2008; Lyytinen et al., 2004). Regarding possible causes of early literacy difficulty, genetic risk of reading difficulties at the single word level currently appears to be similar across countries studied (Byrne et al., 2002; Paulesu et al., 2001), though environmental variables may vary. In studies carried out in English language contexts, for example, lower socio-economic status and learning English as an additional language are commonly cited as risk factors for early literacy difficulties (Noble, Wolmetz, Ochs, Farah, & McCandliss, 2006; Snow Burns & Griffin, 1998). Within the Norwegian context studied here, however, economic deprivation is not a commonly reported reason for low literacy achievement and within the group performing below the national threshold at the end of grade 1, children who spoke another language at home were not more likely to score below the national thresholds in reading and spelling. Chi squared tests instead found that gender was a more important factor: of the variables measured in this study it was boys and children with parents self-reporting RWD who were significantly more likely to score below the national thresholds in word reading and spelling (see Supplementary material, Appendix 2). Gender differences in literacy performance, favouring girls, have been reported in previous studies (e.g. Wei, Liu, & Barnard-Brak, 2015), though this result confirms how early this gap may emerge. It will be important that future studies also explore the possible remedial implications of the relationship between rhythm processing and literacy skills. As well as Overy’s rhythm based intervention described above (Overy, 2000), Flaugnacco and colleagues (Flaugnacco, Lopez, Terribili, Montico, Zoia, & Schön, 2015) recently carried out a randomized control trial of children with developmental dyslexia (mean age 10 years), implementing a 30-week musical intervention alongside conventional treatment (n = 24) in comparison to an art plus conventional treatment control group (n = 22). The musical intervention, based on Kodaly and Orff pedagogy, had a strong rhythm focus. At follow-up, while both groups made reading progress, the music group had made significantly greater gains in reading accuracy, and phonological processing skills as compared to the art control group. Bhide, Power and Goswami (2013) in a smaller scale study, but one that included a similar age group to that reported here (6–7 year olds), and included tapping activities as an intervention very similar to the assessment included here, also reported that a rhythm focused intervention had similarly positive effects on literacy skills as a computerized literacy-focused programme over a period of approximately 2 months. Thus, future studies should combine early detection of reading risk, including the measurement of rhythm skills, with an accompanying rhythm-focused intervention.

Another outstanding question for the field is the relationship between speech and non-speech rhythm sensitivity, and their respective links to literacy. In the introduction to this study, a hypothesized relationship was outlined between non-speech rhythm, phonological processing and literacy. Phonological processing in its broadest definition includes sensitivity to speech rhythm, or prosody—the stress, rhythm and intonation of speech (Hayes, 1995; Selkirk, 1980) and both speech and non-speech rhythm have been demonstrated to have predictive relationships to literacy (Goswami, Gerson, & Astruc, 2010; Wade-Woolley & Heggie, 2016; Wood & Terrell, 1998). Assessment of speech rhythm necessarily adds language demands to a task, and in order to try to assess speech rhythm specifically, many existing tasks heavily implicate meta-linguistic skill (see Wade-Woolley & Heggie, 2016, for a review), complicating the interpretation of predictive models. In this study we were specifically interested in children’s untrained rhythm skill, and its predictive power independent of wider language development. However, within the growing research field exploring links between rhythm and literacy, an increased integration of knowledge concerning assessment and intervention across both speech and non-speech rhythm domains is imperative.

## Conclusion

As mentioned in the introduction, many predictors of early reading ability have already been identified (Melby-Lervåg, Lyster, & Hulme, 2012; Scarborough, 1998). However, finding the optimal combination of measures, to yield levels of classification accuracy that would justify the financial costs of intervening early and minimize the emotional costs of misidentification, remains an international challenge. In this study, inclusion of a measure of rhythmic timing alongside more commonly used measures significantly increased the classification accuracy of predicting children who would be struggling at the end of grade 1. The correct identification rate of poor spelling abilities increased with 9.2% points when adding a simple measure of rhythmic timing.

The use of rhythmic timing measures with young children also offers some distinct advantages. Because the task is relatively free of language demands, it may provide new ways of predicting difficulties in reading and spelling for children being educated in a language that is different to that used at home. It is also quick to administer, with the digital presentation allowing for standardized usage by a variety of educational personnel. Subsequent work by our research group is following the literacy development of the same group of children as they progress further in their school careers.

## Electronic supplementary material

Below is the link to the electronic supplementary material.

Supplementary material 1 (DOCX 17 kb)
