# Supplementary material for: Rhythm production at school entry as a predictor of poor reading and spelling at the end of first grade
Source: Read Writ. 2017 Oct 23;31(1):215–37. doi: 10.1007/s11145-017-9782-9 (PMC5752745; doi:10.1007/s11145-017-9782-9)
Supplement: Supplementary file 1 — Supplementary material 1 (DOCX 17 kb) [file 11145_2017_9782_MOESM1_ESM.docx]

Supplementary Material: Appendix A

*Correlations among predictors.*

|  | Gender | Fam. RWD | STM | Voc. | LK | F. ph. | Blend. | RAN | 1.5 Hz |
| --- | --- | --- | --- | --- | --- | --- | --- | --- | --- |
| Gender |  | 0.07 | 0.11* | 0.03 | 0.05 | 0.16 | 0.13** | -0.02 | -0.04 |
| FamRWD |  |  | -0.09* | -0.02 | -0.10* | -0.14** | 0.10* | 0.14** | 0.09* |
| STM |  |  |  | 0.35** | 0.25** | 0.35** | 0.33** | -0.24** | -0.16** |
| Voc. |  |  |  |  | 0.32** | 0.46** | 0.39** | -0.24 | -0.28** |
| LK |  |  |  |  |  | 0.52** | 0.39** | -0.25** | -0.22** |
| F. ph. |  |  |  |  |  |  | 0.53** | -0.27** | -0.24** |
| Blend. |  |  |  |  |  |  |  | -0.28** | -0.25** |
| RAN |  |  |  |  |  |  |  |  | 0.20** |
| 1.5 Hz |  |  |  |  |  |  |  |  |  |

*Note*. **p*<.05, ***p*<.01

Supplementary Material: Appendix B

1. Results of Chi-Square Test for independence and percentages of girls vs. boys and children with and without RD-parent(s) who score below thresholds in reading and spelling at the end of grade 1 (n = 479).

|  | Girl | Boy | χ^2^ | *p* | Phi | RWD in fam. | No RWD in fam. | χ^2^ | *p* | Phi |
| --- | --- | --- | --- | --- | --- | --- | --- | --- | --- | --- |
| Read below threshold | 9.7 | 17.6 | 5.67 | 0.02 | -0.12 | 25.0 | 10.7 | 11.41 | 0.00 | 0.16 |
| Spell below threshold | 9.7 | 18.0 | 6.29 | 0.01 | -0.12 | 29.5 | 10.0 | 21.82 | 0.00 | 0.22 |

1. Results of Chi-Square Test for independence and percentages of children with and without Scandinavian home language who score below thresholds in reading and spelling at the end of grade 1 (n = 479).

|  | Scandinavian  home lang. | Not Scan.  home lang. | χ^2^ | *p* | Phi |
| --- | --- | --- | --- | --- | --- |
| Read below threshold | 11.9 | 22.1 | 4.34 | 0.04 | -0.10 |
| Spell below threshold | 11.7 | 25.0 | 7.73 | 0.01 | -0.14 |
